# Core autophagy genes and immune infiltration characteristics in rheumatoid arthritis: A bioinformatics study

**DOI:** 10.1371/journal.pone.0326168

**Published:** 2025-07-11

**Authors:** Zining Peng, Qian Deng, Yuanbo Huang, Fanyu Meng, Yuan Long, Yuanyuan Wei, Weitian Yan, Xiaoyu Zhang, Jiangyun Peng, Zhaofu Li, Nian Liu

**Affiliations:** 1 The First School of Clinical Medicine, Yunnan University of Chinese Medicine, Kunming, Yunnan, PR China; 2 Department of Rheumatology, The No.1 Affiliated Hospital of Yunnan University of Chinese Medicine/Yunnan Provincial Hospital of Traditional Chinese Medicine, Kunming, Yunnan, PR China; 3 Yunnan Key Laboratory of Dai and Yi Medicines, Yunnan University of Chinese Medicine, Kunming, Yunnan, PR China; Hong Kong Baptist University, HONG KONG

## Abstract

**Objective:**

This study aims to identify autophagy-related biomarkers in rheumatoid arthritis (RA) synovium, analyze their immune infiltration characteristics, and validate therapeutic potential through multi-level experimental approaches.

**Methods:**

We used public datasets to obtain synovial tissue genes of healthy people and RA patients, screened differentially expressed genes (DEGs) of RA, and intersected with the human autophagy gene database (HADb) to obtain RA autophagy genes. GO and KEGG enrichment analysis and single-gene genome enrichment analysis were performed. The diagnostic value of RA core autophagy genes in the validation set was screened and verified. The immune cell correlation analysis of RA autophagy core genes was performed to obtain the correlation between single disease autophagy core genes and immune cells. Finally, we prepared CIA rat models to verify the autophagy protein P62, Beclin-1 and the 11 core genes associated with RA-autophagy.

**Results:**

A total of 1098 RA DEGs were obtained. Intersecting with 222 autophagy genes obtained from the HADb database yielded 27 RA autophagy genes. GO analysis of RA autophagy genes showed 307 biological mechanisms. KEGG enrichment analysis obtained 86 signaling pathways, including FoxO, Necroptosis and other pathways related to RA autophagy. GSEA analysis found that the control group had a higher correlation with adipokine signaling pathways and others. And 11 RA autophagy-related core genes (IFNG, EGFR, MYC, CXCR4, MAPK8, CASP1, TNFSF10, CTSB, FAS, FOXO1, FOXO3) were obtained by screening the PPI network, and there were differences in expression in the training set (*P* < 0.001). External validation set verification showed diagnostic efficacy. Analysis of immune infiltration in RA autophagy-related genes revealed 14 immune cell types differentially abundant in synovial tissues of RA patients vs. normal controls. Significant correlations exist between autophagy genes and immune subsets. Finally, animal experiments showed that joint autophagy was enhanced (*P* < 0.001), and the mRNA of 11 RA-autophagy core genes had significant changes (*P* < 0.001).

**Conclusion:**

We systematically identified 11 autophagy-related core genes as potential therapeutic targets for RA. Our CIA model validation provides preclinical evidence supporting their translational potential. These genes showed significant correlations with 14 synovial immune cell subtypes, may serve as novel therapeutic targets by modulating immune infiltration and inflammatory pathways. Future investigations should focus on elucidating the mechanistic basis of the observed gene-immune cell interactions in both autophagic and immune pathways to facilitate the development of precision therapies.

## 1. Introduction

Rheumatoid arthritis (RA) is a disease mainly manifested by joint swelling, tenderness, and synovial inflammation in small joints such as proximal interphalangeal joints and metacarpophalangeal joints. If it persists and is not cured, bone destruction may occur in the later stage [1]. Epidemiological investigations show that the global prevalence of RA is about 0.21% − 1.0%. In a study based on the UK Clinical Practice Research Datalink, more than 22 million adults over 20 years old were included. It was found that the incidence of most autoimmune diseases including RA showed an upward trend during the study period [2,3]. Given the absence of curative therapies, early intervention remains critical for achieving disease remission.

The pathogenesis of RA is extremely complex and has not been fully elucidated. It is a multi-factor and multi-step immune-mediated inflammatory process involving the interaction of multiple levels such as genetic susceptibility, environmental factors, abnormal activation of the immune system, and autoimmune reactions [4]. RA pathogenesis involves dysregulated activation of immune cells (e.g., macrophages, T cells) and inflammatory molecules. Other immune cells such as macrophages, dendritic cells, natural killer cells, as well as the complement system and cytokine network also jointly participate in the pathological process of RA [5]. Programmed cell death forms such as apoptosis, autophagy, necroptosis, and pyroptosis play an important role in the pathophysiology of RA. Among them, autophagy, as a process of cellular self-degradation, still needs in-depth study on its specific mechanism of action in RA [6].

Autophagy is a stress response generated by cells themselves. Under stress, cells transport denatured, damaged, and non-functional proteins and subcellular organelles to lysosomes for degradation or recycling to maintain their own structural, functional, and metabolic stability [7]. However, excessive autophagy or insufficient autophagy will trigger autophagic stress, induce damage to cell structure, and then cause continuous cell damage [8]. Autophagy plays dual roles in RA, it maintains cellular homeostasis by clearing damaged organelles (survival mechanism), yet excessive autophagy may trigger programmed cell death pathways, exacerbating joint damage. Most exosomes in RA are produced by white blood cells and synovial cells and can promote citrullinated proteins, inflammatory molecules, and RA-related enzymes. Autophagy, as a trigger for post-translational modification of proteins and extracellular vesicles, may be an effective factor in preventing inflammation in RA patients [9,10]. In addition, the role of autophagy in the pathogenesis of RA includes citrullination, breakdown of immune tolerance, osteoclastogenesis, dysplasia of RA-FLS cells, resistance to apoptosis, and the therapeutic potential of autophagy regulators [11,12]. Therefore, in-depth analysis of RA-related autophagy core genes and immune infiltration characteristics can not only improve the complex pathological progression of RA but also provide new targets for personalized treatment strategies related to RA autophagy pathways and immune infiltration.

**Fig 1:**
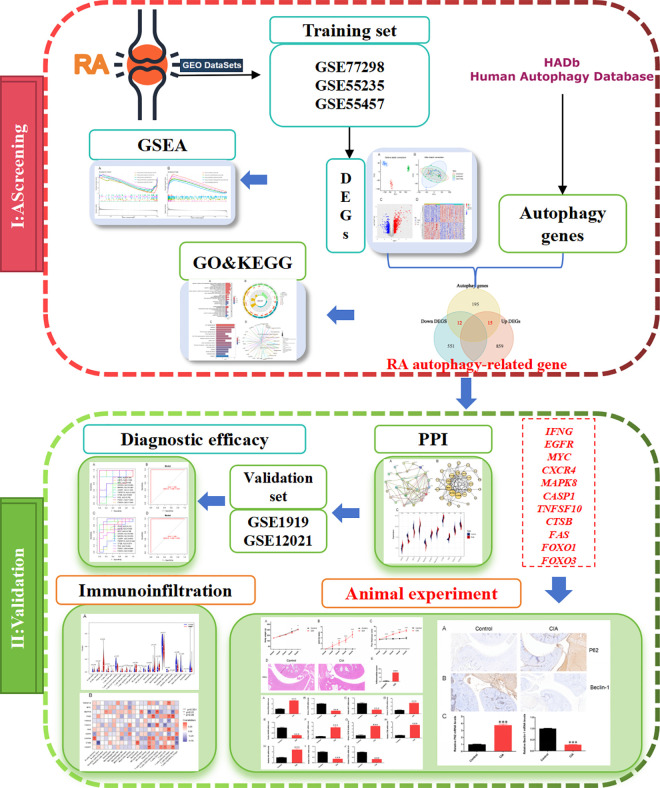
Research flow chart of this study.

In this study, we first obtained gene chip data of multiple groups of synovial tissue samples from RA patients and healthy controls from the Gene Expression Omnibus (GEO) database and performed differential expression analysis. After obtaining the results, cross the autophagy-related genes with the differentially expressed RA genes, perform GO and KEGG analysis, and single-gene genome enrichment analysis. After screening the core genes related to RA and autophagy, verify the diagnostic value in the synovial tissue of RA patients and perform immune cell correlation analysis of the core genes. Finally, we prepared CIA rat models to verify the autophagy protein P62, Beclin-1 and the 11 core genes associated with RA-autophagy. The workflow of this study is shown in Fig 1.

## 2. Materials and methods

### 2.1 Acquisition and differential analysis of RA datasets

Through the GEO database (https://www.ncbi.nlm.nih.gov/geo/), we retrieved RA-related GEO datasets using “rheumatoid arthritis” as the search term. Gene probes in the raw data were annotated using the chip platform’s annotation file, and probes not matching any gene name were excluded. For genes detected by multiple distinct probes, the mean value of these probes was selected as the final expression level of the gene. After quantile normalization was applied to the training set data to eliminate technical variations between microarrays, the sva package’s ComBat algorithm was employed for batch correction to remove systematic batch effects across datasets. The effectiveness of batch effect removal was verified by principal component analysis (PCA). Subsequently, RA-related differentially expressed genes (DEGs) were screened based on the criteria of |log2FC| > 0.585 and adj. *P* < 0.05.

### 2.2 Screening of RA autophagy-related genes

The autophagy-related gene set is derived from the human autophagy database (HADb) (http://www.autophagy.lu/). Intersect DEGs with autophagy genes to obtain RA autophagy genes.

### 2.3 GO and KEGG enrichment analysis

Using the “Bioconductor” software package of R (v4.3.0), perform GO and KEGG enrichment analysis on RA autophagy genes according to *P <*  0.05. GO enrichment analysis includes biological process (BP), cellular component (CC), and molecular function (MF). KEGG enrichment analysis further explores the signaling pathways they are enriched in.

### 2.4 Single-gene genome enrichment analysis

To evaluate the potential biological pathway changes of RA autophagy gene-related biomarkers, single-gene genome enrichment analysis (gene set enrichment analysis, GSEA) is performed by using the “enrichplot” software package of R (v4.3.0). “c2.cp.kegg.symbols.gmt” is regarded as the reference genome.

### 2.5 Screening of RA autophagy core genes

Construct a protein-protein interaction network (PPI) of RA autophagy genes through the STRING (https://cn.string-db.org/) website. Set the species to “Homosapiens”, set the minimum interaction threshold to the highest confidence “highest confidence (0.4)”, and hide free nodes. Then apply Cytoscape (v 3.9.1) to calculate the degree value of this network, and obtain the core genes of the topological network according to the degree value. Use the “ggplot2” and “ggpubr” packages of R software to draw violin plots of the expression of RA autophagy characteristic genes in the training set.

### 2.6 Diagnostic value of core autophagy genes in the validation set

Normalize the external GSE1919 and GSE12021 datasets, respectively, to construct a disease-control model validation set to evaluate the degree of association between RA autophagy core genes and diseases. Use the “pROC” package to draw the ROC curve and use the area under the curve (AUC) to evaluate the diagnostic efficacy of autophagy core genes.

### 2.7 Immune cell correlation analysis of RA autophagy core genes

Use R software to analyze the corrected gene expression matrix of the training set obtained above through the CIBERSORT algorithm, screen out samples with *P* < 0.05, and use the “vioplot” software package to compare the joint synovium of the screened RA samples and normal samples, and analyze each immune cell. The level of immune infiltration difference between the two groups, *P* < 0.05 means that the difference between the two groups is significant.

### 2.8 Animal experiment

#### 2.8.1 Chemicals and reagents.

Bovine type II collagen (#20021) was purchased from Chondrex, Inc. (Woodinville, USA). Incomplete Freund’s adjuvant (IFA) (#KX0210047Q-10) and Complete Freund’s adjuvant (FA) (#KX0210046Q-10) were acquired from Biodragon (Suzhou, China). Glacial acetic acid (#20181227) was purchased from Tianjin Zhiyuan Chemical Reagent Co. HE staining kits (#G1076) were purchased from Wuhan Servicebio Technology Co., Ltd. (Wuhan, China). TRIzol (#15596018) was purchased from Inv itrogen (USA), HiScript III RT SuperMix for qPCR (+gDNA wiper) (#R323-01) and Taq Pro Universal SYBR qPCR Master Mix (#Q712-02) were purchased from Vazyme Biotech Co.,Ltd (Nanjing, China), Immunohistochemical kit (#G1215-200T), Anti-Beclin 1 antibody (#GB115741) and Anti-p62 antibody (#GB11531) were purchased from Wuhan Servicebio Technology Co., Ltd. (Wuhan, China).

#### 2.8.2 Experimental animals and groups.

Wistar male rats (6−7 weeks old, weighing 180-220g) were purchased from SPF Biotechnology Co., Ltd. (Beijing, China) and the animal license number is SCXK (Jing) 2019−0010. The rats were raised at a suitable temperature (25°C) with a 12 h light/dark cycle. All animal experiments were approved bythe Ethics Committee of Yunnan Provincial Hospital of TCM, animal experiments conform to the “3R” principle and the ethics approval number was DW2025−003. The CIA model (CIA, n = 5) was established based on the previous report and improved it [13]. Bovine type II collagen was emulsified with equal volume of CFA on ice. Rats were injected subcutaneously with 0.2 ml of a pre-prepared emulsion at the base of the tail, except for the control group (Control, n = 5), and on day 7 CFA was replaced with IFA and 0.1 ml of emulsion was injected in the same manner for boosted immunization. Control group rats were injected with equal volume of saline. From the first immunization, the rats were scored with reference to the arthritis Index (AI) and paw thickness every 7 days until the end of the 28th and the rats were killed.

#### 2.8.3 Hematoxylin and eosin (HE) staining.

Following tissue harvesting, rat joint specimens were either paraffin-embedded or cryopreserved. Paraffin-embedded tissues underwent sequential deparaffinization using xylene substitutes I and II (15 min each), followed by dehydration in graded ethanol series (100% I/II/III: 5 min each; 75%: 2 min) and rinsing in distilled water. Fresh-frozen sections were equilibrated at −20°C, thawed at room temperature, fixed in methanol (1 min), and air-dried to prevent OCT compound interference, while cell monolayers were fixed with 4% paraformaldehyde (5 ~ 10 min) and rinsed. All sections were pretreated with a high-definition staining enhancer (1 min), stained with hematoxylin (3 min), differentiated (3 ~ 5 s), and blued (1 min) with optimized reagents, followed by eosin Y counterstaining (15 ~ 30 s). Dehydration through graded ethanol (95%: 60 s; 100% I/II/III: 2 min each), clearing in xylene (2 min), and mounting with neutral resin. Preceded whole-slide digital scanning at 100 × magnification.

#### 2.8.4 Immunohistochemical staining.

Paraffin-embedded ankle joint sections from control and CIA rats were deparaffinized, rehydrated, and subjected to antigen retrieval using citrate buffer (pH 6.0, 95°C, 20 min). Endogenous peroxidase activity was quenched with 3% H₂O₂ in methanol (10 min), followed by blocking with 10% normal goat serum (37°C, 30 min). Sections were incubated overnight at 4°C with primary antibodies against P62 and Beclin-1 (1:1000), then treated with HRP-conjugated secondary antibodies. DAB was applied for 30–60 s for chromogenic detection, counterstained with hematoxylin, and mounted with neutral resin. For quantitative analysis, preceded whole-slide digital scanning at 100 × magnification.

#### 2.8.5 Real-time PCR.

Synovial tissues from rat joints were homogenized in RNA lysis buffer using sterile, RNase-free consumables. Total RNA was extracted by pulverizing 5–20 mg tissues in pre-chilled tubes with 3 mm grinding beads and 1 mL RNA lysis buffer, followed by centrifugation (12,000 × g, 10 min, 4°C). Supernatants were mixed with chloroform substitute, incubated, and centrifuged to separate phases. The aqueous phase was precipitated with isopropanol at −20°C for 15 min, centrifuged, and the RNA pellet was washed twice with 75% ethanol, air-dried, and resuspended in RNase-free water. Reverse transcription was conducted using 10 μL RNA, 5 × SweScript SuperMix, gDNA Remover, and nuclease-free water (20 μL total), with thermal cycling at 25°C (5 min), 42°C (30 min), and 85°C (5 sec). qPCR reactions (15 μL/sample) comprised 7.5 μL SYBR Green Master Mix, 1.5 μL primers (2.5 μM), 2 μL cDNA, and 4 μL water, amplified on a real-time PCR system with cycling conditions: 95°C (30 s), 40 cycles of 95°C (15 s)/60°C (30 s), and melt curve analysis (65–95°C, 0.5°C increments). Gene expression was quantified via the ΔΔCT method, with primer sequences provided in Supplementary S1 Table.

#### 2.8.6 Statistical analysis.

All data were expressed as mean ± standard deviation (SD) and analyzed using SPSS 26.0 software. Data conforming to normal distribution were tested by two independent samples t test. A *p*-value < 0.05 was considered statistically significant.

## 3. Results

### 3.1 Grouping and expression of GEO datasets

**Fig 2:**
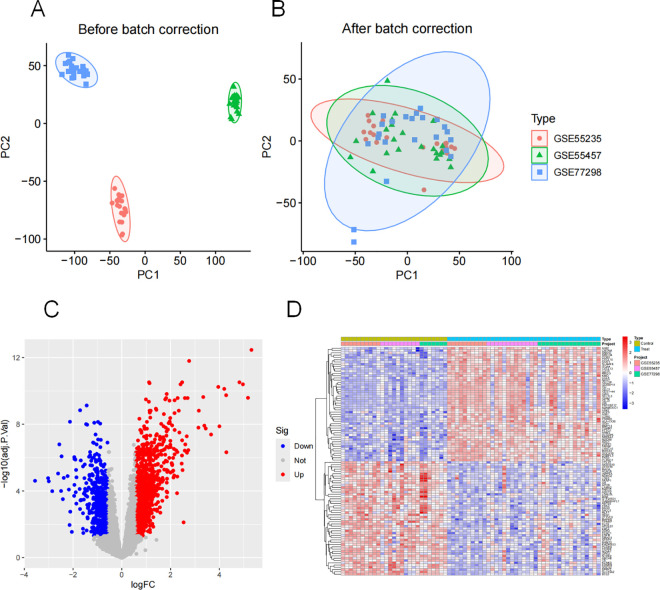
Expression of the training set after batch correction and correction. Note: (A-B). principal component analysis (PCA) of gene expression level before and after batch treatment; (C). Digs expresses volcano map; (D). The first 20 upward and downward DEGs.

GSE77298, GSE55235, and GSE55457 were used as the training set, while GSE1919 and GSE12021 were used as the external validation sets. The gene data of the training set were merged and batch-corrected (Fig 2A and 2B). According to the screening conditions, 1098 DEGs were obtained (Fig 2C), including 563 downregulated genes and 874 upregulated genes. Fig 2D shows the top 50 upregulated and downregulated DEGs. The detailed information of the gene chip is presented in Table 1.

**Table 1:** Basic information and grouping of GEO data sets.

GEO ID	Platform file	Control group	Trial group	Groups	Tissue source
**GSE77298**	GPL570	7	16	Training set	Synovial tissue
**GSE55235**	GPL96	10	10		
**GSE55457**	GPL96	10	13		
**GSE1919**	GPL91	5	5	Validation set	
**GSE12021**	GPL96	9	12		

### 3.2 Autophagy genes of RA

**Fig 3:**
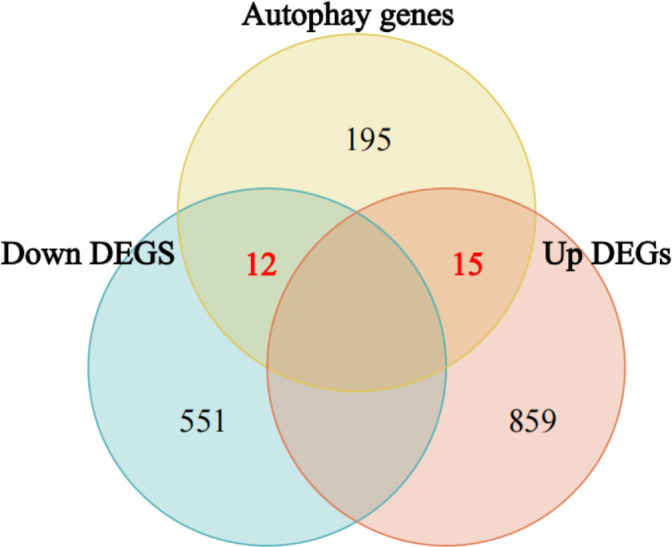
Venn diagram of intersection of DEGs and autophagy genes.

222 autophagy genes were obtained from the HADb database. The intersection of DEGs and autophagy genes yielded 27 RA autophagy genes, among which there were 15 upregulated autophagy genes and 12 downregulated autophagy genes (Fig 3).

### 3.3 Enrichment analysis

**Fig 4:**
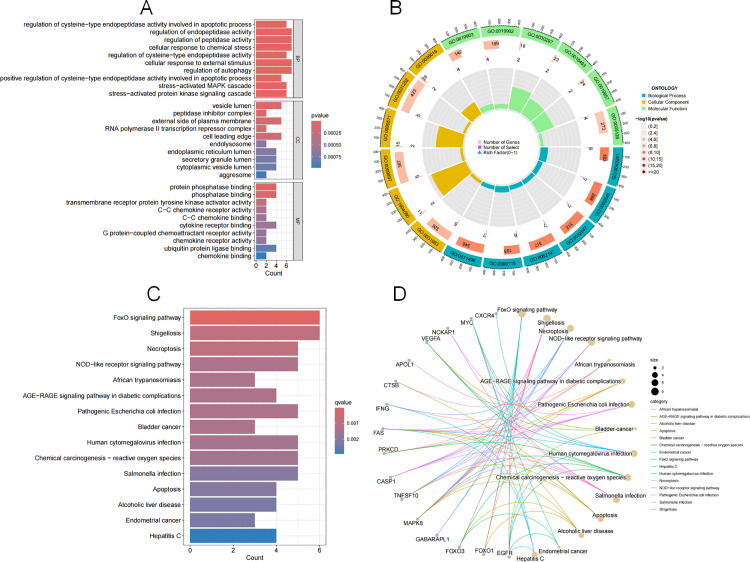
GO enrichment analysis and KEGG pathway enrichment. Note: (A) GO enrichment bubble diagram, where the horizontal axis of columns BP, CC and MF represents the number of enriched genes in each project, and the size of bubbles represents the number of enriched genes; (B) The outer circle of the diagram represents biological processes, yellow represents cell components, green represents molecular processes, and the inner circle represents the size of the P value. The darker the color, the smaller the P value; (C) KEGG enrichment histogram. The horizontal axis represents the overall ratio of the function of the gene, and the vertical axis represents the enriched pathway. The color represents the degree of expression of P based on correction, and the redder the acceptance, the more significant the enrichment; (D) KEGG network analysis. The circle on the left represents genes that are pathway enriched; The yellow circle in the upper right represents the enriched KEGG signaling pathway. The circle size represents the number of core targets currently contained in the KEGG signaling pathway.

The GO analysis of the 27 RA autophagy genes revealed 307 biological mechanisms, mainly through 263 biological processes such as regulation of cysteine-type endopeptidase activity involved in apoptotic process, regulation of endopeptidase activity, and regulation of peptidase activity; they were involved in 13 molecular functions, including protein phosphatase binding, phosphatase binding, and transmembrane receptor protein tyrosine kinase activator activity, etc.; and they were mainly located in 31 cellular components, such as vesicle lumen, peptidase inhibitor complex, and external side of plasma membrane, etc. (Fig 4A and 4B).

A total of 86 related signaling pathways were obtained through KEGG enrichment analysis, among which those related to RA autophagy included FoxO signaling pathway, Necroptosis, NOD-like receptor (NLR) signaling pathway, Apoptosis, AGE-RAGE signaling pathway in diabetic complications, etc. (Fig 4C and 4D).

### 3.4 GSEA analysis

**Fig 5:**
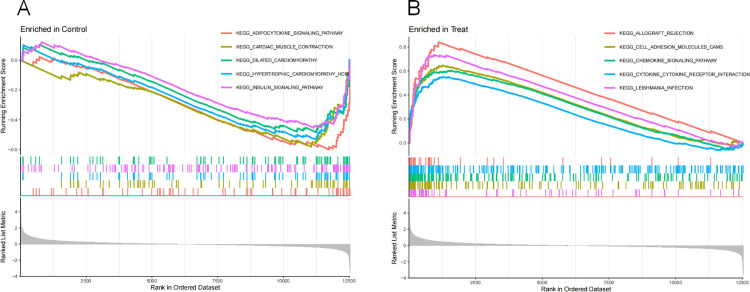
GSEA analysis in the normal group and the RA group in the training set.

The KEGG enrichment of the samples involved in GSEA indicated that the control group had a higher correlation with adipocytokine signaling pathway, cardiac muscle contraction, dilated cardiomyopathy, hypertrophic cardiomyopathy hcm, insulin signaling pathway (Fig 5A), while pathways such as allograft rejection, cell adhesion molecules cams, chemokine signaling pathway, cytokine cytokine receptor interaction, leishmania_infection were enriched in the RA group (Fig 5B).

### 3.5 RA autophagy core genes and their expression

**Fig 6:**
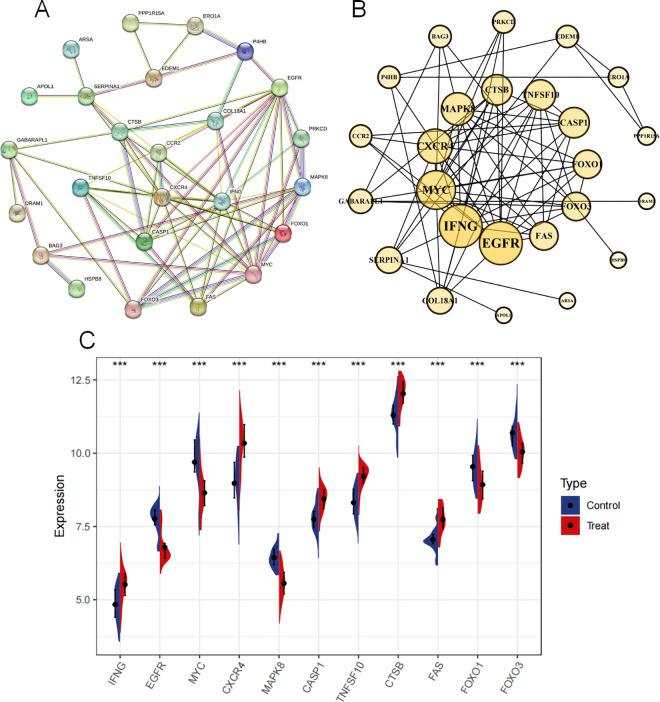
Screening and expression of RA autophagy core genes. Note: (A). Protein interaction network diagram; (B).cotoscape visualized the PPI network and distributed the size according to the degree value; (C). RA autophagy core gene in the training set. ***P < 0.001.

The PPI network was visualized using Cytoscape v.3.8.0. The network contained 59 nodes and 486 edges (Fig 6A and 6B). Based on the degree value of the topological network, IFNG, EGFR, MYC, CXCR4, MAPK8, CASP1, TNFSF10, CTSB, FAS, FOXO1, FOXO3 were obtained, these 11 genes are the core genes related to RA autophagy, and these degree values are ranked in Table 2. According to Fig 6C, in the training set, the expressions of IFNG, CXCR4, CASP1, TNFSF10, CTSB, FAS were upregulated, while the others showed a downward trend, and all the expressions were significantly different (*P* < 0.001).

**Table 2:** Ranking the top PPI network degree values.

Name	Gene Symbol	Degree
**Interferon gamma**	IFNG	14
**Epidermal Growth Factor Receptor**	EGFR	14
**Myc Proto-Oncogene Protein**	MYC	12
**C-X-C Chemokine Receptor Type 4**	CXCR4	10
**Mitogen-Activated Protein Kinase 8**	MAPK8	9
**Caspase 1**	CASP1	8
**Tumor Necrosis Factor (Ligand) Superfamily, Member 10**	TNFSF10	8
**Cathepsin B**	CTSB	8
**TNF Receptor Superfamily, Member 6**	FAS	7
**Forkhead Box O1**	FOXO1	7
**Forkhead Box O3**	FOXO3	7

### 3.6 Verification of the diagnostic ability of RA core autophagy genes

**Fig 7:**
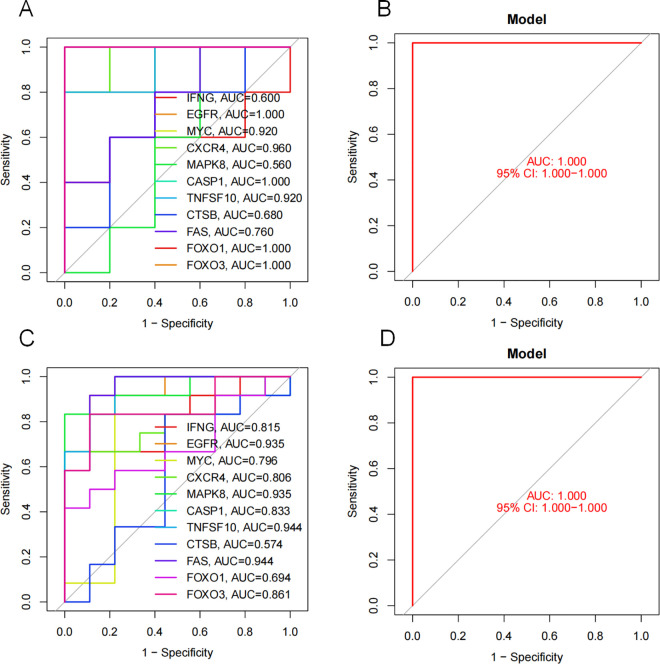
ROC curve of RA autophagy core gene in validation set. Note: (A). Verification of the accuracy of core genes in external validation set GSE1919; (B). Verification of the accuracy of core genes in GSE12021.

The obtained IFNG, EGFR, MYC, CXCR4, MAPK8, CASP1, TNFSF10, CTSB, FAS, FOXO1, FOXO3 were externally verified using two external validation sets. The area under the ROC curve (AUC) > 0.5, indicating that these core genes have certain diagnostic efficacy in predicting RA (Fig 7).

### 3.7 Immune infiltration analysis of RA autophagy core genes

**Fig 8:**
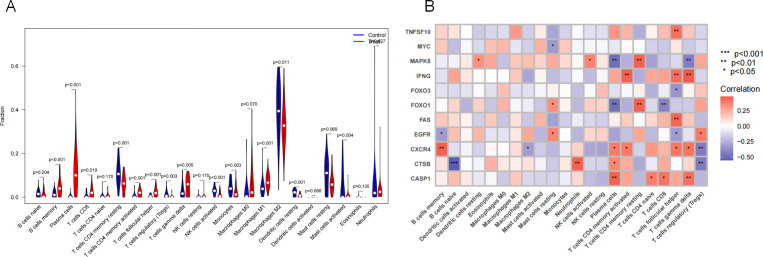
Immune infiltration analysis of RA autophagy core genes. Note: (A). The difference in the proportion of immune cells in the training set; (B). Correlation between autophagy core genes and immune cells.

The corrected gene chip expression levels of the training set were analyzed using the CIBERSORT algorithm. Compared with the joint synovium of normal individuals, there were significant differences in 14 types in the joint synovium of RA patients, including B cells memory, plasma cells, CD8 T cells, resting CD4 T cells memory, activated CD4 T cells memory, follicular helper T cells, regulatory T cells (Tregs), γδT cells, activated natural killer cells, monocytes, M1 macrophages, M2 macrophages, resting dendritic cells, and activated mast cells (Fig 8A).

By comparing the correlations between individual disease autophagy core genes and immune cells, TNFSF10 was positively correlated with T cells follicular helper. MYC was negatively correlated with Mast cells resting. MAPK8 was positively correlated with Dendritic cells resting, NK cells activated, T cells CD4 memory resting, and negatively correlated with Plasma cells, T cells gamma delta. IFNG was positively correlated with T cells CD4 memory activated, T cells follicular helper, T cells gamma delta. FOXO3 was negatively correlated with T cells follicular helper. FOXO1 was positively correlated with Mast cells resting, T cells CD4 memory resting, and negatively correlated with Plasma cells, T cells CD8T cells CD8. FAS was positively correlated with T cells follicular helper. EGFR was positively correlated with Mast cells resting, T cells regulatory (Tregs), and negatively correlated with B cells memory, T cells follicular helper. CXCR4 was positively correlated with B cells memory, Plasma cells, T cells CD4 memory activated, T cells follicular helper, T cells gamma delta, and negatively correlated with Macrophages M2, T cells regulatory (Tregs). CTSB was positively correlated with Neutrophils, Plasma cells, and negatively correlated with B cells naive, Tregs. CASP1 was positively correlated with Plasma cells, T cells CD4 naive, T cells CD8, T cells gamma delta. (Fig 8B).

### 3.8 Animal experiment results

#### 3.8.1 The general condition of CIA rats.

**Fig 9:**
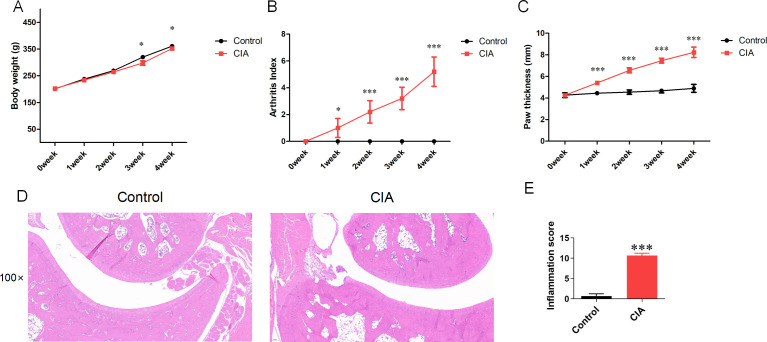
The general condition of CIA rats. Note: (A). Changes of body weight in Control group and CIA group at 0-4 weeks; (B). Changes of AI scores in Control group and CIA group in 0-4 weeks; (C). Changes of paw thickness in Control group and CIA group in 0-4 weeks; (D). HE staining of rat joints; (E). Inflammation scores of HE staining; Compared with the Control group, *P < 0.05, ***P < 0.001.

Over the course of the experimental weeks, all rats exhibited an increase in body weight, with the control group gaining the most and the CIA group gaining the least (*P *< 0.05) (Fig 9A). To evaluate the efficacy of CIA model rats, paw thickness and arthritis scores (0–8 points) were measured. Both metrics were significantly elevated in the CIA group compared to the control group by week 4 (*P* < 0.05) (Fig 9B and 9C). HE staining results demonstrated that the joints of rats in the CIA group exhibited prominent inflammatory cell infiltration and bone destruction. Compared with the Control group, the joint space in the CIA group was markedly narrowed, and the inflammation score was significantly elevated (*P *< 0.001) (Fig 9D and 9E).

#### 3.8.2 Expression of autophagy gene P62 and Beclin-1 in CIA rats.

**Fig 10:**
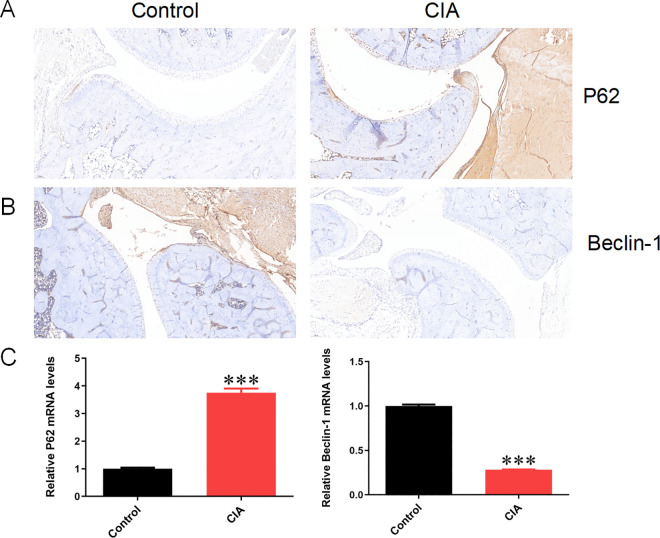
Expression of autophagy gene P62 and Beclin-1 in CIA rats. Note: (A). Immunohistochemical staining of P62 in the joint synovial tissues of Control and CIA rats; (B). Immunohistochemical staining of Beclin-1 in the joint synovial tissues of Control and CIA rats; (C). The mRNA expression of P62 and Beclin-1 in the joint synovial tissues of Control and CIA rats; Compared with the Control group, *P < 0.05, ***P < 0.001.

Immunohistochemical staining of autophagy proteins P62 and Beclin-1 was performed on the joints of Control and CIA rats. It was found that there was less positive staining of P62 and more staining of Beclin-1 positive brown granules in the joints of rats in the control group. Compared to control rats, in the joint staining of rats in the CIA group, the staining of P62 positive brown granules significantly increased, and Beclin-1 significantly decreased (Fig 10A and 10B). Consistent with the results of immunohistochemical staining, mRNA detection of joint synovial tissues revealed that the mRNA expression of P62 in the joint synovial tissues of rats in the Compared to control rats and CIA group increased, while the expression of Beclin-1 decreased (*P *< 0.001) (Fig 10C).

#### 3.8.3 Expression of mRNA of core genes in CIA rats.

**Fig 11:**
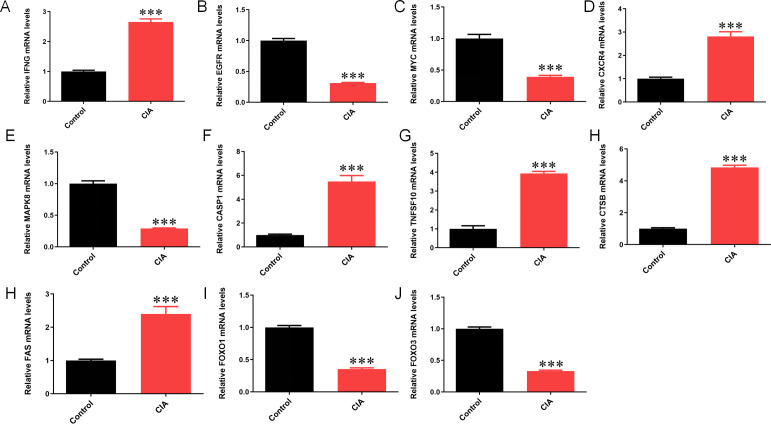
Expression of mRNA of core genes in CIA rats. Note: ***P < 0.001.

The mRNA detection of core genes in the joint synovial tissues of Control and CIA rats was conducted, as shown in the following figure. Compared to control rats, mRNA expression of RA autophagy-related core genes *IFNG, CXCR4, CASP1*, *TNFSF10, CTSB* and *FAS* were significantly elevated, while *EGFR, MYC, MAPK8, FOXO1* and *FOXO3* expression was reduced in the synovial tissues (*P *< 0.001) (Fig 11). These results are consistent with the result expression of the RA autophagy core genes in the training set.

## 4. Discussion

Currently, the utilization of public databases and bioinformatics analysis techniques for identifying disease pathogenesis, autophagy core factors and their biological functions, as well as exploring related factors, constitutes an active domain in bioinformatics research [13]. In this study, we exploited GEO datasets and autophagy – related databases to identify RA – autophagy – related biomarkers and infer the immune infiltrating cells, thereby analyzing and clarifying their potential roles in the progression of RA.

We obtained 27 RA autophagy genes by intersecting the DEGs of RA with autophagy genes, among which 15 were upregulated and 12 were downregulated. For these 27 genes, GO and KEGG analyses were conducted. The results demonstrated that autophagy mainly modulates biological processes such as regulation of cysteine – type endopeptidase activity involved in apoptotic process, regulation of endopeptidase activity, and regulation of peptidase activity, along with molecular functions including protein phosphatase binding, phosphatase binding, and transmembrane receptor protein tyrosine kinase activator activity. Moreover, it is located in cellular components such as vesicle lumen, peptidase inhibitor complex, and external side of plasma membrane, amounting to a total of 307 biological mechanisms. KEGG enrichment analysis revealed that the main pathways related to RA autophagy are distributed in FoxO signaling pathway, Necroptosis, NOD – like receptor signaling pathway, Apoptosis, AGE – RAGE signaling pathway in diabetic complications, etc.

Firstly, the involvement of the FoxO signaling pathway implies its critical regulatory role in RA autophagy. As an important transcription factor, the FoxO protein participates in various physiological processes of cells, including autophagy [14]. FoxO may promote the formation of autophagosomes, enhance the clearance of damaged organelles and inflammation – related proteins, thereby attempting to maintain intracellular environment stability. However, under the abnormal state of RA, this regulation may be disrupted. Activating the FoxO1 signaling transduction in RA – FLS can modulate the levels of autophagy, proliferation, invasion, migration, and pro – inflammatory factors within the disease [15]. The enrichment of the Necroptosis pathway presents a new perspective for RA autophagy research. Generally, autophagy is considered a cell survival mechanism. However, its association with Necroptosis suggests that autophagy may be involved in the regulatory network of programmed necrosis in RA [16]. In the joint tissues of RA, the inflammatory microenvironment may trigger Necroptosis, and during this process, autophagy may attempt to alleviate the damage caused by Necroptosis. Alternatively, there may be a complex interaction of mutual promotion or inhibition between the two, influencing the fate of joint synovial cells and chondrocytes and thereby altering the progression of RA [17].

The NLR signaling pathway reveals autophagy’s role in regulating the immune-inflammatory response of RA. NLRs recognize pathogen-associated molecular patterns (PAMPs) and damage-associated molecular patterns (DAMPs), initiating inflammatory cascades, while autophagy interacts with this pathway to modulate immune cell responses to joint abnormalities [18]. Additionally, the interplay between autophagy and apoptosis in RA is critical: autophagy protects joint cells from excessive apoptosis under normal conditions, but its dysfunction (either impaired or hyperactivated) may accelerate apoptosis-driven joint destruction, highlighting the need to balance these processes in therapeutic targeting [19]. Furthermore, the enrichment of the advanced glycation end product-receptor for AGEs (AGE-RAGE) pathway, a key player in diabetic complications, suggests shared inflammatory-metabolic mechanisms between RA and diabetes [20]. Autophagy may mitigate AGE-RAGE-induced oxidative stress by clearing AGE-modified proteins or regulating RAGE expression, linking RA pathogenesis to metabolic inflammation [21]. Collectively, these pathways provide mechanistic insights into RA autophagy, offering novel directions for understanding disease progression and developing targeted therapies.

KEGG enrichment via GSEA revealed that the control group showed stronger associations with pathways critical to metabolic and cardiovascular homeostasis, including adipocytokine signaling, cardiac muscle contraction, and insulin signaling. These findings highlight the importance of baseline physiological mechanisms in deciphering disease pathogenesis. Comparative analysis of these pathways between healthy and RA states provides mechanistic clues for disease progression and therapeutic targeting. PPI network analysis identified 11 RA-autophagy core genes (*IFNG, EGFR, MYC, CXCR4, MAPK8, CASP1, TNFSF10, CTSB, FAS, FOXO1, FOXO3*). External validation demonstrated their diagnostic potential, with ROC curve AUC values >0.5, supporting their utility in RA prediction and subsequent immune infiltration studies. In addition, our animal studies have confirmed that autophagy expression is enhanced in CIA rat models. Compared to control rats, mRNA expression of RA autophagy-related core genes *IFNG, CXCR4, CASP1*, *TNFSF10, CTSB* and *FAS* were significantly elevated, while *EGFR, MYC, MAPK8, FOXO1* and *FOXO3* expression was reduced in the synovial tissues. These results further validate the conclusions and feasibility of this study.

In this study, through the analysis of the corrected gene chip expression levels of the training set using the CIBERSORT algorithm, it was found that compared with normal individuals, there were significant differences in 14 types of immune cells in the joint synovium of RA patients. These immune cell types likely play a crucial role in the pathogenesis of RA. The alterations in B – cell memory, plasma cells, etc., suggest that humoral immunity has been significantly affected during the pathological process of RA. The change in the number of B – cell memory cells may imply the continuous recognition and response of the immune system to antigens in the joint synovium, while the abnormal number of plasma cells may be associated with the excessive production of autoantibodies that can attack joint tissues, triggering inflammation and damage [22].

The changes in T – cell subsets such as CD8 T cells, CD4 T cells in different states, follicular helper T cells, and regulatory T cells are also of great significance. CD8 T cells play a role in killing target cells in the immune response, and their variations in the synovium of RA patients may suggest an abnormal immune – killing effect on joint synovium cells [23]. The imbalance of CD4 T – cell subsets in different states may affect cytokine secretion, thereby regulating the direction and intensity of the immune response. For example, the change in the memory of activated CD4 T cells may enhance the inflammatory response, while the abnormal number or function of regulatory T cells may lead to the breakdown of immune tolerance, allowing the autoimmune response to persist [24]. In addition, the differences in immune cells such as γδ T cells, activated natural killer cells, monocytes, M1 and M2 - type macrophages, resting dendritic cells, and activated mast cells cannot be ignored [25,26]. γδ T cells have a unique role in local tissue immunity, and their abnormalities in the RA synovium may be involved in the initiation and maintenance of local inflammation. The change in the activation state of natural killer cells may affect their immune surveillance and killing ability towards diseased synovium cells. M1 - type macrophages usually participate in the pro – inflammatory response, while M2 - type macrophages are associated with tissue repair. The imbalance between the two may exacerbate inflammation and tissue damage [27].

Our immune correlation analysis revealed distinct regulatory patterns between autophagy-related genes and immune subsets in RA pathogenesis. TNFSF10 and FAS exhibited positive associations with follicular helper T cells (Tfh), implicating their roles in aberrant humoral immunity via germinal center regulation. Conversely, MYC and EGFR displayed negative correlations with resting mast cells and plasma cells, respectively, suggesting regulatory suppression of inflammatory mediator release. MAPK8 demonstrated dual functionality, showing positive links to antigen-presenting dendritic cells and NK cells but negative associations with plasma cells and γδ T cells, indicative of its balance between immune surveillance and inflammatory restraint. Notably, CXCR4 exhibited pan-immune modulation, positively correlating with B/T cell memory subsets while negatively associating with regulatory T cells and M2 macrophages, potentially shaping synovial immune cell trafficking. The FOXO family displayed divergent effects: FOXO3 inversely correlated with Tfh, whereas FOXO1 promoted resting mast cell/CD4 + memory states. Pro-inflammatory drivers IFNG and CASP1 showed consistent activation profiles across T cell subsets and plasma cells, while CTSB synergistically enhanced neutrophil/plasma cell activity while suppressing naïve B cell tolerance. These multilayered interactions collectively orchestrate RA synovial inflammation through coordinated regulation of immune activation thresholds and effector cell polarization.

Our findings on autophagy-related core genes and their immune infiltration characteristics in RA provide novel insights that complement and extend previous studies in this field. Notably, Fan et al. identified 52 autophagy-related genes and validated 10 hub genes (e.g., CASP8, CTSB, MYC) through bioinformatic analyses and qRT-PCR validation in clinical samples [28]. While their work pioneered the exploration of autophagy-related biomarkers in RA, our study advances this field by integrating multi-omics approaches to reveal dynamic interactions between autophagy genes and immune infiltration—a critical aspect not addressed in their research. Specifically, we identified 11 core autophagy genes (IFNG, EGFR, CXCR4, etc.), seven of which (64%) were distinct from their reported hub genes, highlighting the discovery of novel RA-autophagy candidates such as IFNG and EGFR. These newly identified genes may reflect the heterogeneity of RA pathogenesis and offer broader therapeutic targeting potential. Furthermore, Li et al. focused on machine learning-based biomarker discovery and immune infiltration patterns but limited their validation to computational models [29]. In contrast, our study bridges the gap between bioinformatic predictions and biological validation by experimentally confirming the dysregulation of all 11 core genes in CIA rat models, including autophagy proteins (P62, Beclin-1) and mRNA changes. This dual validation strategy strengthens the reliability of our findings compared to purely computational approaches. Additionally, while Zhang et al. identified IRF4 as a single autophagy-related diagnostic biomarker through functional experiments in MH7A cells, our work systematically unravels the network of autophagy-immune crosstalk by linking multiple core genes (e.g., FOXO1/3) to 14 distinct immune cell subsets, including macrophages and dendritic cells—a dimension unexplored in their study [30]. In addition to existing bioinformatic studies on RA-autophagy, which inherently present certain limitations, our research seeks to build upon this foundation and address these critical gaps [31].

Despite these findings, our study has several limitations that warrant consideration. Firstly, while CIBERSORT analysis provided valuable insights into immune cell infiltration patterns, its resolution is inherently constrained by bulk RNA-seq data, which may obscure subtle differences between closely related immune subsets and underestimate rare cell populations. Secondly, although we identified 11 autophagy-related core genes and validated their expression in CIA models, the direct mechanistic links between these genes and RA pathogenesis remain incompletely resolved. Thirdly, while our core gene panel includes novel candidates like IFNG and EGFR not reported in earlier studies, the therapeutic potential of these targets needs rigorous validation through functional experiments. Finally, reliance on public GEO datasets introduces potential batch effects, and our CIA model, though widely used, may not fully recapitulate the heterogeneity of human RA. Integrating multi-omics approaches and validating findings across diverse cohorts could strengthen the clinical relevance of these findings. These limitations underscore the need for mechanistic studies in RA and clinical translation of autophagy-targeted therapies.

## 5. Conclusion

In summary, the correlations between these autophagy core genes and immune cells provide a novel perspective for in – depth understanding of the pathogenesis of RA. These genes may be involved in the complex immune – inflammatory process in the joint synovium of RA by regulating the functions, numbers, and interactions of immune cells. Future research could further explore the mechanism of action of these genes in immune cells and investigate whether targeting these genes or their associated signal pathways can be used for RA treatment.

## Supporting information

S1 Table Primer sequence information. (DOCX)
