# Supplementary material for: Core autophagy genes and immune infiltration characteristics in rheumatoid arthritis: A bioinformatics study
Source: PLoS One. 2025 Jul 11;20(7):e0326168. doi: 10.1371/journal.pone.0326168 (PMC12250634; doi:10.1371/journal.pone.0326168)
Supplement: S1 Table — (DOCX) [file pone.0326168.s001.docx]

**S1 Table. Primer sequence information**

| Primer name | Primer sequence (5'-3') | bp |
| --- | --- | --- |
| R-GAPDH-S | CTGGAGAAACCTGCCAAGTATG | 138 |
| R-GAPDH-A | GGTGGAAGAATGGGAGTTGCT |  |
| R-IFNG-S | GGTGAACAACCCACAGATCCAG | 96 |
| R-IFNG-A | CTTTTCCGCTTCCTTAGGCTAG |  |
| R-Egfr-S | AGAACAACACCCTGGTCTGGAA | 180 |
| R-Egfr-A | CCACCACTACTATGAAGAGGAGGC |  |
| R-MYC-S | TGGAGGAGACATGGTGAATCAG | 96 |
| R-MYC-A | AAGCCGCTCCACATACAGTCC |  |
| R-CXCR4-S | GTGGGCAATGGGTTGGTAAT | 262 |
| R-CXCR4-A | CGTGGACAATGGCAAGGTAG |  |
| R-Mapk8-S | TCGTTACTACAGAGCACCAGAGG | 168 |
| R-Mapk8-A | GGTGTTCCGAGCTGTTCAATAA |  |
| R-Casp1-S | TGTAATGAAGACTGCTACCTGGC | 251 |
| R-Casp1-A | CGAGTGGGTGTTTTCATTATTGG |  |
| R-TNFSF10-S | CTTCAGTCAGCACTTCACGATG | 119 |
| R-TNFSF10-A | CCTGTAGCTGTTTCACCTCGTTG |  |
| R-ctsb-S | GCTGGACGCAACTTCTACAATG | 112 |
| R-ctsb-A | GATTTATGTCCTCGCTGAACCC |  |
| R-fas-S | ACACGAACCAGCAACACCAAAT | 156 |
| R-fas-A | AGGACTTGGGATTCCAGATTCA |  |
| R-FOXO1-S | CTTCAAGGATAAGGGCGACAG | 299 |
| R-FOXO1-A | GCCATTTAGAAAACTGAGACCCA |  |
| R-Foxo3-S | AACAGTACCGTGTTCGGACC | 119 |
| R-Foxo3-A | AGTGTCTGGTTGCCGTAGTG |  |
| R-beclin1-S | AGGAGTTGCCGTTGTACTGTTCT | 178 |
| R-beclin1-A | GTGTCTTCAATCTTGCCTTTCTCC |  |
| R-P62-S | GCTATTACAGCCAGAGTCAAGGG | 105 |
| R-P62-A | TGGTCCCATTCCAGTCATCTT |  |

Note. R: Rat; Identification of upstream primers: -S (sense primer); Identification of downstream primers: -A (antisense primer).
